# Conical Gradient Junctions of Dendritic Viologen Arrays on Electrodes

**DOI:** 10.1038/srep11122

**Published:** 2015-06-09

**Authors:** Takehiro Kawauchi, Yuki Oguchi, Keiji Nagai, Tomokazu Iyoda

**Affiliations:** 1 Iyoda Supra-Integrated Material Project, Exploratory Research for Advanced Technology (ERATO), Japan Science and Technology Agency (JST), and Frontier Research Center, Tokyo Institute of Technology, 4259-S2-3 Nagatsuta, Midori-ku, Yokohama 226-8503, Japan

## Abstract

The three-dimensional construction of arrays of functional molecules on an electrode surface, such as organic semiconductors and redox-active molecules, is a considerable challenge in the fabrication of sophisticated junctions for molecular devices. In particular, well-defined organic layers with precise molecular gradients are anticipated to function as novel metal/organic interfaces with specific electrical properties, such as a space charge layer at the metal/semiconductor interface. Here, we report a strategy for the construction of a three-dimensional molecular array with an electrical connection to a metal electrode by exploiting dendritic molecular architecture. Newly designed dendritic molecules consisting of viologens (1,1′-disubstituted-4,4′-bipyridilium salts) as the framework and mercapto groups as anchor units form unique self-assembled monolayers (SAMs) on a gold surface reflecting the molecular design. The dendritic molecules exhibit a conical shape and closely pack to form cone arrays on the substrate, whereas, in solution, they expand into more flexible conformations. Differences in the introduction position of the anchor units in the dendritic structure result in apical- and basal-type cone arrays in which the spatial concentration of the viologen units can be precisely configured in the cones. The concentration in apical-type SAMs increases away from the substrate, whereas the opposite is true in basal-type SAMs.

Dendritic architecture, which is often observed in living organisms, plays critical roles in fundamental functions for life and growth. For instance, the dendrites of the neuron are branching neurites that extend conically from the cell body to increase surface area^1^. In a neural network, neurons are connected to each other at this increased surface through robust multiple synapse formation. Such a conical structure is also observed in the retina of the eyes. Cone cells, which are one of the photoreceptors in the retina, contain a conical outer segment that includes photopigments. These cone cells are closely packed together with rod cells to form the photoreceptor layer on the pigmented epithelium, in which all basal faces of the conical outer segments are directed toward the incident light^2^. Inspired by the spatial control, robust multiple connections, signal transmission, and formation of unique assembled layers observed in neurons, we were motivated to develop a new molecular-assembled system based on dendritic architecture. Dendritically structured molecules composed of redox units as the framework organized on a substrate under spatial control result in three-dimensional arrays of the functional units. Here, we report the first construction of three-dimensional molecular arrays with controlled gradient structures on gold surfaces by utilizing self-assembly of dendritic viologen-arranged molecules with mercapto groups at the apex and periphery of the dendritic structure.

## Results

**Figure 1:**
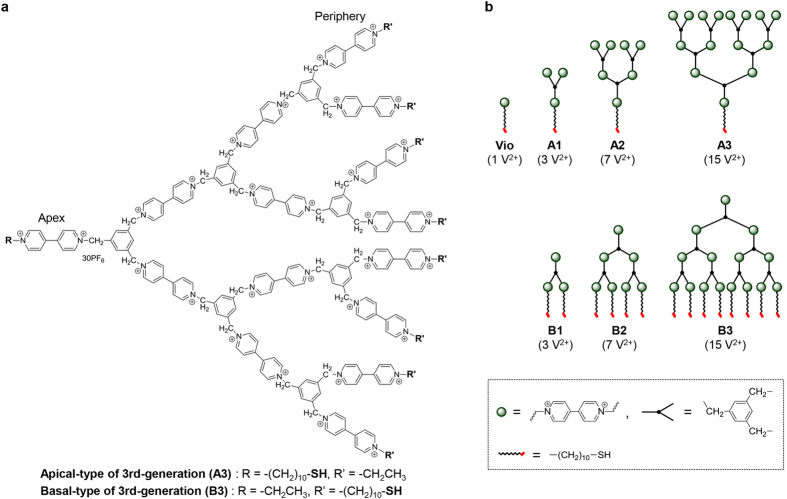
(**a**) Chemical structure of the 3rd-generation dendritic viologen-arranged molecule. (**b**) Schematic of **Vio**, apical- and basal-type molecules. The number of viologen units (V^2+^) is indicated in parentheses.

**Figure 2:**
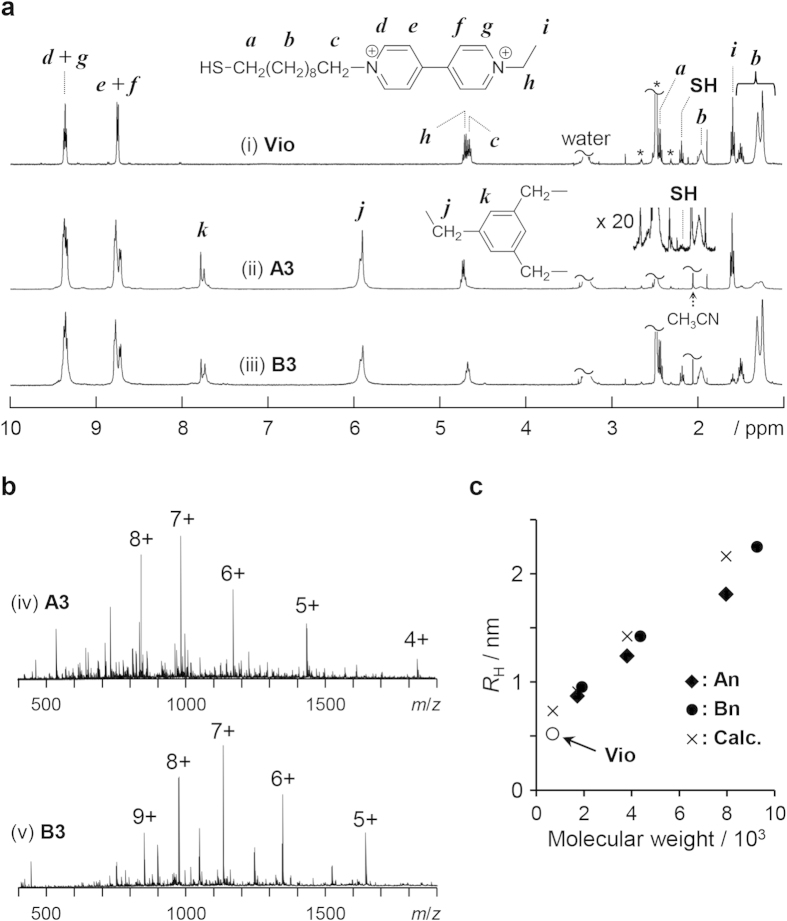
Characterization of the viologen-arranged molecules. (**a**) ^1^H NMR spectra of **Vio**, **A3** and **B3** measured in DMSO-*d*_6_. The asterisks denote DMSO-*d*_5_ and its ^13^C satellite signals. (**b**) Positive mode ESI-MS spectra of **A3** and **B3**. See Supplementary Figs. S2 and S3. (**c**) Hydrodynamic radii (*R*_H_s/nm) of acetyl-capped **Vio**, **An**, and **Bn** (n = 0–3) determined by NMR in deuterated acetonitrile at 25 °C as a function of molecular weight. The calculated radii of gyration (*R*_calc_) estimated by MM calculation for acetyl-capped **Vio** and **An** are also plotted. See Supplementary Table S1 and Fig. S4.

The chemical structure of these designed molecules is shown in Fig. 1. Viologens, 1,1′-disubstituted-4,4′-bipyridilium salts, are well known to function as redox-active molecules and as electron acceptors^3^. The viologen units are connected by benzene rings through a methylene tether not only to facilitate electron transport between the viologen units via a through-bond hopping mechanism but also to reduce molecular mobility due to the shortest alkyl chain^4^,^5^,^6^,^7^,^8^,^9^,^10^. To self-assemble the molecules onto a gold surface, ω-mercaptodecyl groups were regioselectively introduced at the apex (apical, **A**) and periphery (basal, **B**) of the dendritic structures. Designed molecules with several generations were successfully synthesized via a microwave-heating technique (Supplementary Scheme S1 and Fig. S1), and their structures were confirmed by elemental analysis, ^1^H NMR, and electrospray ionization mass (ESI-MS) measurements (see Methods). Fig. 2a shows the assigned ^1^H NMR spectra of the compound consisting of one viologen unit (**Vio**) and 3rd-generation dendritic molecules (**A3** and **B3**). In the ^1^H NMR spectrum of **A3** (ii), the intensity of the signal corresponding to the mercapto group was considerably lower than expected, probably because of encapsulation within the dendritic structure. Direct evidence for the introduction of the mercapto group was obtained in the ESI-MS spectrum, which contained a series of signals assigned to the ionized species of **A3** (iv in Fig. 2b).

To evaluate the size of the dendritic molecules in solution, the diffusion coefficients (*D*s) were determined using acetyl-capped dendritic molecules before a deprotection reaction (acetyl-capped **Vio**, **An,** and **Bn**, n = 1–3) via a diffusion NMR experiment in which the sample was dissolved in deuterated acetonitrile (Supplementary Table S1). The hydrodynamic radii (*R*_H_s) of acetyl-capped **Vio**, **A3**, and **B3** were calculated to be 0.52, 1.81, and 2.25 nm, respectively, on the basis of the *D* values according to the Stokes equation. The *R*_H_ values increased with increasing dendritic generation (Fig. 2c). The *R*_H_-to-generation ratios for **An** and **Bn** were 0.47 and 0.65 nm/generation, respectively; these values are in fairly good agreement with the calculated radii of gyration estimated by MM calculations (0.63 nm/generation for acetyl-capped **An**). Note that the peripheral viologen units are extended in the solution.

We then prepared self-assembled monolayers (SAMs) of the dendritic molecules with ω-mercaptodecyl groups on gold substrates. The molecules were dissolved in a mixture of acetonitrile and ethanol (1/1, v/v) at a concentration of 2 mM, and gold substrates were immersed into the solutions. After equilibration for 48 h, the modified substrates were rinsed with acetonitrile, dried under flowing nitrogen, and subjected to electrochemical measurements.

**Figure 3:**
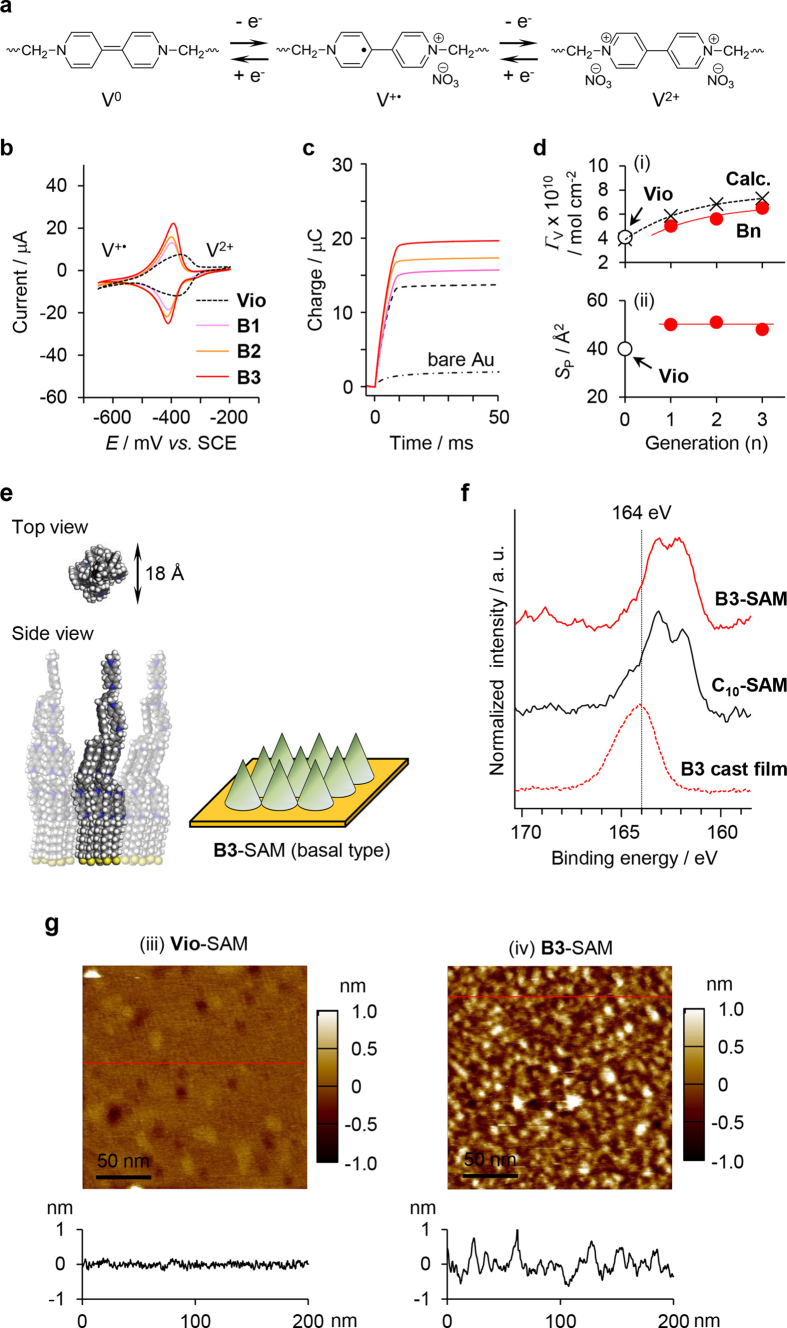
Characterization of basal-type cone arrays prepared with dendritic viologen-arranged molecules with anchor units at their periphery. (**a**) Redox process of the viologen unit. (**b**) Cyclic voltammograms of **Vio**- and **Bn**-SAMs measured in 100 mM NaNO_3_ at a scan rate of 200 mV s^−1^. (**c**) Charge-time curves of bare Au substrate, Vio- and **Bn**-SAMs obtained by chronocoulometry with the potential step from −650 mV to −200 mV vs. SCE. (**d**) Changes in *Γ*_V_ (i) and *S*_P_ (ii) of **Bn**-SAMs (red dots) as a function of dendritic generation. *Γ*_V_ and *S*_P_ values expected under the assumption of a close-packed structure of the peripheral units are also plotted (crosses). (**e**) Space-filling model and schematic of the cone array for **B3**-SAM on the electrode. (**f**) S 2p XPS spectra of B3-SAM, a **B3** cast film, and a typical SAM of C_10_-SH (C_10_-SAM). (**g**) Tapping-mode AFM height images of Vio- (iii) and **B3**-SAMs (iv) observed in 100 mM NaNO_3_. The height profiles along the red lines in the images are also shown.

Viologens exist in three oxidation states through a two-step redox process (Fig. 3a)^11^. Consequently, the viologen units incorporated in the SAM on the gold substrate can be monitored by cyclic voltammetry (CV) using the substrate as an electrode. Figure 3b shows CV profiles of **Vio**- and **Bn**-SAMs recorded in 100 mM aqueous NaNO_3_ and reveals a redox wave at approximately −400 mV vs. SCE corresponding to *E*_1/2_(V^2+^/V^+•^) of the viologen units. In addition, the peak current density of the SAMs prepared with the dendritic molecules was proportional to the scan rate in the range from 50 to 500 mV/s in the CV measurement (Supplementary Fig. S5), indicating that the surface wave behavior resulted from the immobilization of the viologen units. The surface density of viologen units (*Γ*_V_) was determined by the amount of the transported charge resulting from the V^2+^/V^+•^ process in chronocoulometric measurement (Fig. 3c). The surface density of the dendritic molecule (*Γ*_D_) and the area occupied by one molecule (*S*_D_) were calculated from *Γ*_V_, as summarized in Table 1. *n*-Alkanethiols bearing one viologen unit at the head group are known to form well-ordered SAMs on gold substrates, in which the viologen units are perpendicularly oriented to organize close-packed structures and the alkyl chains are loosely packed^12^,^13^,^14^,^15^. The *S* value of **Vio**-*S*AM was estimated to be 40 Å^2^ (Table 1), which agrees with the values reported for the aforementioned viologen SAM (38 Å^2^)^12^,^13^,^14^,^15^ and is substantially larger than that of a conventional *n*-alkanethiol SAM (21.4 Å^2^)^13^,^16^.

Next, we discuss the structure of the SAMs obtained from the dendritic viologen-arranged molecules. With increasing dendritic generation, the redox current of **Bn**-SAMs in the CVs increased (Fig. 3b), resulting in an increase of *Γ*_V_ (i in Fig. 3d). Additionally, the *S*_D_ value increased exponentially with the dendritic generation, as shown in Table 1. The **Bn** molecules are multidentate compounds bearing plural ω-mercaptodecyl groups at the periphery of their dendritic structure. Thus, the area occupied by one ω-mercaptodecyl group, *i.e*., the area occupied by one peripheral viologen unit (*S*_P_), was estimated from the *S*_D_ value. The *S*_P_ value of 50 Å^2^ for **B1**-SAM is consistent with the *S* value of **Vio**-SAM (40 Å^2^), indicating that the peripheral viologen units form a well-organized structure similar to the close-packed structure of the **Vio**-SAM. Interestingly, the *S*_P_ value did not change even when the generation was increased (ii in Fig. 3d), demonstrating that the eight peripheral units of **B3** also form a closely packed structure on the substrate, whereas the other viologen units stack pyramidically on the peripheral layer. When the peripheral viologen units form a close-packed structure similar to the **Vio**-SAM, the redox current should increase with dendritic generation because of the viologen units stacked on the peripheral units. The increasing tendency of *Γ*_V_ was consistent with the change expected from the assumptions (i in Fig. 3d). Figure 3e illustrates a plausible model of **B3**-SAM based on these results, where the **B3** molecules should organize into a cone array anchored at the basal position and the viologen units should stack pyramidically. Analysis by x-ray photoelectron spectroscopy (XPS) provided strong evidence for the model. The key point is that all the ω-mercaptodecyl groups of **B3** are anchored onto the gold substrate through the formation of S-Au bonds. Figure 3f shows the XPS spectrum of the S 2p region for **B3**-SAM, together with the corresponding spectra of a **B3** cast film and a SAM of 1-mercaptodecane (C_10_-SAM) for reference. The sulfur atoms bound to gold were observed at 162.0 and 163.2 eV attributing to 2p_3/2_ and 2p_1/2_, respectively, in the SAMs^17^,^18^. A peak at 164.0 eV observed in the spectrum of the **B3** cast film was assigned to free thiol (-SH)^18^,^19^. The spectrum of **B3**-SAM shows little contribution from free thiol, similar to that of the well-characterized C_10_-SAM, which confirms that all the bundled ω-mercaptodecyl groups of **B3** were bonded together to anchor onto the gold substrate. In general, multidentate adsorbates have been designed to restrict the conformational arrangements of all the anchoring sites^18^,^20^. The **B3** molecule is more flexible than previously reported multidentate adsorbates, as evident from the aforementioned solution behavior. Instead, the molecular conformation was fixed to form a conical shape by anchoring all the ω-mercaptodecyl groups to the gold substrate. As a result, a density gradient of the viologen units was achieved on the electrode. Tapping-mode AFM observations of **Vio**- and **B3**-SAMs in 100 mM NaNO_3_ were carried out to investigate the surface morphology. The AFM height image of **Vio**-SAM revealed a flat and smooth surface with 0.11 nm of RMS roughness, which shows the close-packed structure (iii in Fig. 3g) expected on the basis of the electrochemical measurements. In contrast, a rather rough surface (RMS = 0.46 nm) with partially aggregated molecules was observed in the AFM image of **B3**-SAM (iv in Fig. 3g), as expected for the surface of the previously described cone array.

**Figure 4:**
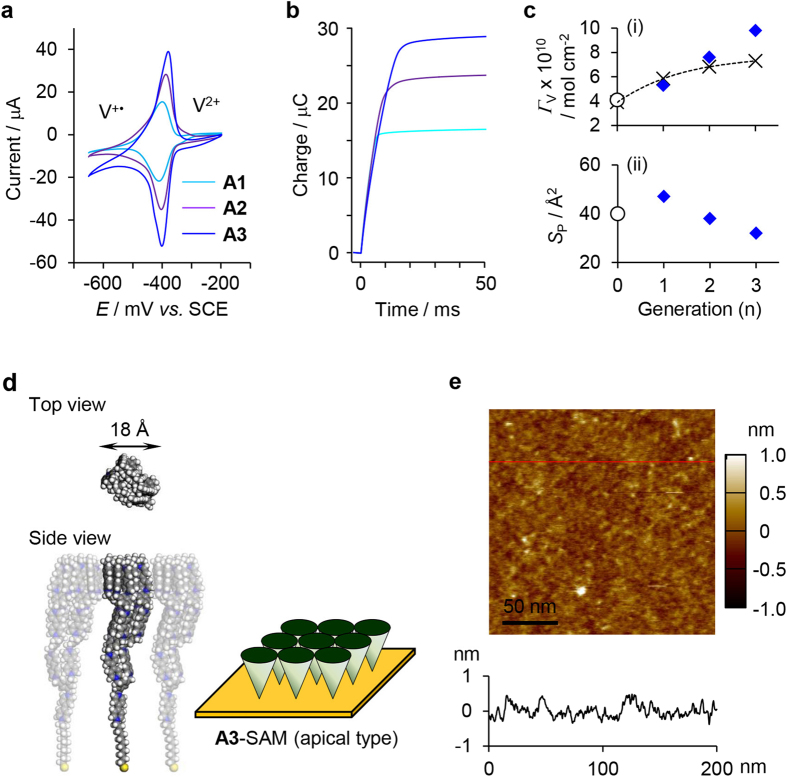
Characterization of apical-type cone arrays prepared with dendritic viologen-arranged molecules with anchor units at their apex. (**a**) Cyclic voltammograms of An-SAMs measured in 100 mM NaNO_3_ at a scan rate of 200 mV s^−1^. (**b**) Charge-time curves of **An**-SAMs obtained by chronocoulometry with the potential step from −650 mV to −200 mV vs. SCE. (**c**) Changes in *Γ*_V_ (i) and *S*_P_ (ii) of **An**-SAMs (blue diamonds) as a function of dendritic generation. *Γ*_V_ and *S*_P_ values expected under the asumption of a close-packed structure of the peripheral units are also plotted (crosses). (**d**) Space-filling model and schematic of the cone array for **A3**-SAM on the electrode. (**e**) Tapping-mode AFM height image of **A3**-SAM observed in 100 mM NaNO_3_. The height profile along the red line in the image is also shown.

We next investigated SAMs prepared with the apical-type (**An**) molecules. The redox current of **An**-SAMs in the cyclic voltammograms increased with increasing dendritic generation (Fig. 4a), indicating that *Γ*_V_ was dominated by the dendritic architecture, similar to the case of **Bn**-SAMs. The *Γ*_V_ values of **An**-SAMs were also determined by the chronocoulometric measurement (Fig. 4b and Table 1). Notably, the increase of *Γ*_V_ observed in **An**-SAMs was similar to that of the model for the close-packed **Bn**-SAMs (i in Fig. 4c), implying the presence of a cone array anchored at the apex (Fig. 4d), which is the reverse configuration of the **Bn**-SAMs. The *Γ*_D_ for **An**-SAMs, *i.e.*, the surface density of the ω-mercaptodecyl group on the electrode, decreased significantly. The *S*_D_ value of 253 Å^2^ for **A3**-SAM was smaller than the cross-sectional molecular area of 506 Å^2^ estimated from the *R*_H_ value of the acetyl-capped **A3** molecule by diffusion NMR, which suggests close-packing of the peripheral units. The area occupied by one peripheral viologen unit (*S*_P_) was estimated to be 32 Å^2^ on the basis of the *S* value of **A3**-SAM (ii in Fig. 4c and Table 1), which is surprisingly similar to the *S* value of **Vio**-SAM (40 Å^2^). From these results, the **A3** molecules are reasonably concluded to adopt a conical shape and organize into a cone array anchored at the apex, where the ω-mercaptodecyl units are sparsely anchored to the substrate but the peripheral viologen units are closely packed at the surface. This structure is the apical-type conical junction illustrated in Fig. 4d. In the AFM height image of **A3**-SAM measured in 100 mM NaNO_3_, a relatively flat surface (RMS = 0.17 nm) was observed because of the closely packed peripheral viologen units (Fig. 4e), unlike **B3**-SAM (iv in Fig. 3g).

By comparison between **An** and **Bn**-SAMs, it was found that the *Γ* values of **An**-SAMs were higher than those of **Bn**-SAMs. The mobility of the peripheral viologen units of the **An** molecules is considered to be relatively high because the peripheral units are remote from the anchor units. Thus, the peripheral units might partially overlap each other in the **An**-SAMs, resulting the higher *Γ* values.

## Discussion

The spatial control based on the conical shape induced by the dendritic structure is often found in supramolecular materials, such as melamine-barbiturate-based rosettes^21^ and discotic liquid crystals^22^,^23^, as well as in neurons. We have demonstrated here that the dendritic molecular architecture can be applied to construct three-dimensional molecular arrays with controlled gradient structures. The dendritic viologen-arranged molecules with ω-mercaptodecyl groups organize into characteristic SAMs that reflect the dendritic structure in which the dendritic molecules have a conical shape and closely pack, resulting in cone arrays on gold substrates. When the ω-mercaptodecyl group is introduced at the periphery of the dendritic structure, the peripheral viologen units form a close-packed layer, whereas the remainder of the viologen units stacks pyramidically on the layer; these pyramidically stacked viologen units provide a basal-type cone array in which the spatial concentration of the viologen units decreases away from the substrate. In contrast and unexpectedly, an apical-type cone array was obtained when the ω-mercaptodecyl group was introduced at the apex of the dendritic structure. In the apical-type SAM, the viologen concentration increases away from the substrate. This structure is reminiscent of the photoreceptor layer in the retina.

Like the dendrites of the neuron, the dendritic viologen-arranged molecules exhibited electron-transporting ability due to the presence of the redox-active viologen units, as evidenced by the electrochemical measurement of their SAMs. Thus, the apical- and basal-type cone arrays on the gold substrates can be considered molecularly structured metal/organic junctions^24^,^25^,^26^,^27^. At the metal/semiconductor junction, the gradient of the space-charge layer formed in the semiconductor layer in close proximity to the metal can be controlled by Fermi-level matching at the different densities of state, resulting in rectification. Therefore, the construction of structured metal/organic junctions on various metal substrates and nanoparticles may provide molecular devices with tunable electrical properties based on the controlled gradient structure^28^,^29^,^30^,^31^,^32^. Work along these lines is currently in progress.

## Methods

### General

Microwave-assisted syntheses were performed using an Anton Paar Monowave-300 microwave reactor. The microwave reactor was operated in a temperature-controlled mode. NMR spectra were measured on an AVANCE III spectrometer (400 MHz for ^1^H, Bruker Corporation, USA) in chloroform-*d* (CDCl_3_) and dimethylsulfoxide-*d*_6_ (DMSO-*d*_6_) at 25 °C for structural analysis and in acetonitrile-*d*_3_ (CD_3_CN) at 25 °C for measurements of diffusion coefficients. Chemical shifts in the ^1^H NMR spectra were referenced to residual CHCl_3_ (7.26 ppm) and DMSO-*d*_5_ (2.49 ppm). Elemental analyses were carried out by the Center for Advanced Materials Analysis at the Tokyo Institute of Technology. Electrospray ionization mass (ESI-MS) spectra were recorded using a micrOTOF II mass spectrometer (Bruker Daltonics, USA). X-ray photoelectron spectra were collected using an ESCA-3400 spectrometer (Shimadzu Corporation, Kyoto, Japan). Electrochemical measurements were performed using an COMPACTSTAT electrochemical analyzer (IVIUM, Florida, USA) in a standard three-electrode system equipped with a SAM-modified gold working electrode, a platinum counter electrode, and a saturated calomel reference electrode (SCE). The area of the working electrode was approximately 0.28 cm^2^. Atomic force microscopy (AFM) observations were performed at ambient temperature using standard silicon cantilevers (normal frequency of 300 kHz, Oxford Instruments, Oxfordshire, UK) and a Cypher S microscope (Oxford Instruments). Molecular modeling and molecular mechanics (MM) calculations were performed on a Windows PC using the COMPASS II force field as implemented in the Materials Studio software (version 7.0, Accelrys, California, USA).

All starting materials and anhydrous solvents were obtained from Wako Pure Chemical Industries (Osaka, Japan), Tokyo Chemical Industry (Tokyo, Japan), and Sigma-Aldrich (USA) and were used without further purification unless otherwise noted. 1-Ethyl-4-(4′-pyridyl)pyridinium hexafluorophosphate (**Et-Py**) was prepared by mixing 4,4′-bipyridine and bromoethane at room temperature, followed by ion exchange of Br^-^ for PF_6_^-^ using an aqueous solution of NH_4_PF_6_^33^. Gold substrates were prepared either by thermal evaporation or physical deposition onto chromium-deposited silicon wafers (Cr layer thickness: approximately 100 Å; Au layer thickness: approximately 500 Å) immediately before use. For the AFM observations, Au substrates deposited onto cleaved mica were purchased from Agilent Technologies (California, USA) and cleaned using a UV/ozone method immediately prior to use.

### Synthesis of 10-(acetylthio)-1-bromodecane (AcS(CH_2_)_10_Br)

The title compound was prepared in 82% yield from 10-bromo-1-decene by thiol-ene addition with thioacetic acid in the presence of 2,2′-azobis(isobutyronitrile) in toluene under reflux according to the procedure reported in the literature^34^. ^1^H NMR (CDCl_3_, 25 °C) *δ:* 1.22–1.48 (br, CH_2_, 12H), 1.56 (quint, CH_2_, *J* = 7.3 Hz, 2H), 1.85 (quint, CH_2_, *J* = 7.2 Hz, 2H), 2.32 (s, SCOCH_3_, 3H), 2.86 (t, CH_2_, *J* = 7.3 Hz, 2H), 3.40 (t, CH_2_, *J* = 6.9 Hz, 2H).

### Synthesis of 1-(10-acetylthiodecanyl)-4-(4′-pyridyl)pyridinium hexafluorophosphate (AcS-Py)

A solution of AcS(CH_2_)_10_Br (2.2 g, 7.45 mmol) in acetonitrile (40 mL) was added via a syringe pump (4 mL/h) to a stirred solution of 4,4′-bipyridine (11.6 g, 74.5 mmol) in acetonitrile (80 mL) at 75 °C. The reaction mixture was further stirred for 4 h at 75 °C and then evaporated to dryness. The crude product was purified by washing with ethyl acetate several times. After the obtained compound was dissolved into a minimum amount of acetonitrile/methanol mixture (1/1, v/v), a saturated aqueous solution of NH_4_PF_6_ was added dropwise. The precipitates were collected by centrifugation, washed with water several times, and then dried under vacuum at 40 °C for 12 h to give 2.9 g of **AcS-Py** as a white powder in 75% yield. ^1^H NMR (DMSO-*d*_6_, 25 °C) *δ:* 1.15–1.36 (br, CH_2_, 12H), 1.46 (quint, CH_2_, *J* = 7.2 Hz, 2H), 1.95 (br, CH_2_, 2H), 2.29 (s, SCOCH_3_, 3H), 2.79 (t, CH_2_, *J* = 7.2Hz, 2H), 4.62 (t, N^+^-CH_2_, *J* = 7.5 Hz, 2H), 8.03 (d, Ar_pyridyl_-H, *J* = 6.1 Hz, 2H), 8.62 (d, Ar_pyridyl_-H, *J* = 6.8 Hz, 2H), 8.86 (d, Ar_pyridinium_-H, *J* = 6.1 Hz, 2H), 9.22 (d, Ar_pyridinium_-H, *J* = 6.8 Hz, 2H).

### Synthesis of acetyl-capped Vio

AcS(CH_2_)_10_Br (250 mg, 0.85 mmol) and **Et-Py** (363 mg, 1.10 mmol) were dissolved in anhydrous acetonitrile (5 mL). After stirring at 80 °C for 12 h under argon, the reaction mixture was poured into a large amount of ethyl acetate to precipitate the crude product, which was collected by centrifugation and washed with ethyl acetate several times. The crude product was purified by dissolving into a minimum amount of acetonitrile/methanol mixture (1/1, v/v), precipitated into ethyl acetate, washed with ethyl acetate, and then dried under vacuum. The obtained product was dissolved into a minimum amount of acetonitrile/methanol again, and a saturated aqueous solution of NH_4_PF_6_ was added dropwise. The precipitated product was collected by centrifugation, washed with water several times, and then dried under vacuum at 40 °C for 12 h to give 300 mg of acetyl-capped **Vio** as a white powder in 51% yield. ^1^H NMR (DMSO-*d*_6_, 25 °C) *δ:* 1.18–1.34 (br, CH_2_, 12H), 1.48 (quint, CH_2_, *J* = 7.3 Hz, 2H), 1.60 (t, CH_3_, *J* = 7.4 Hz, 3H), 1.96 (br, CH_2_, 2H), 2.30 (s, SCOCH_3_, 3H), 2.80 (t, CH_2_, *J* = 7.3 Hz, 2H), 4.58–4.76 (q + t, N^+^-CH_2_, 4H), 8.68–8.82 (m, Ar_pyridinium_-H, 4H), 9.28–9.42 (m, Ar_pyridinium_-H, 4H). ESI-MS: *m*/*z* = 545.22 [M + PF_6_]^+,^ 200.13 [M]^2+^. Anal. Calcd for C_24_H_36_F_12_N_2_OP_2_S: C, 41.74; H, 5.25; N, 4.06; S, 4.64. Found: C, 41.44; H, 5.03; N, 4.02, S, 4.93.

### Synthesis of basal-type dendritic viologen-arranged molecules

The viologen-arranged molecules with ω-mercaptodecyl groups were prepared via a microwave-heating technique^35^. The synthetic route of **B3** is shown in Supplementary Scheme S1, and the procedures are described below. The reactions were monitored by ^1^H NMR analysis, as shown in Supplementary Fig. S1.

### Synthesis of B1-Br

A solution of **Et-Py** (300 mg, 0.91 mmol) and TBMB (3.24 g, 12.6 mmol) in acetonitrile (15 mL) was heated at 110 °C for 5 min by microwave irradiation. After cooling to room temperature, the reaction mixture was condensed into a small portion under reduced pressure and then poured into a large amount of toluene. The precipitates were collected by centrifugation and washed with toluene several times. The collected compound was purified by being dissolved in a minimum amount of acetonitrile/methanol mixture (1/1, v/v), precipitated into ethyl acetate, washed with ethyl acetate and then dried under vacuum. After the compound was dissolved into a minimum amount of acetonitrile/methanol again, a saturated aqueous solution of NH_4_PF_6_ was added dropwise to precipitate the product, which was collected by centrifugation and washed with water. The compound was purified by being dissolved in acetonitrile, precipitated into water, washed with water, and then dried under vacuum at 40 °C for 12 h to give 592 mg of **B1-Br** in 86% yield. ^1^H NMR (DMSO-*d*_6_, 25 °C, see Fig. S1b) *δ:* 1.59 (t, CH_3_, *J* = 7.3 Hz, 3H), 4.64–4.76 (s + q, Br-CH_2_ and N^+^-CH_2_, 6H), 5.93 (s, N^+^-CH_2_-Ar, 2H), 7.58–7.65 (m, Ar-H, 3H), 8.68–8.83 (m, Ar_pyridinium_-H, 4H), 9.33–9.52 (m, Ar_pyridinium_-H, 4H).

### Synthesis of B1-Py

A solution of **B1-Br** (385 mg, 0.51 mmol) and 4,4′-bipyridine (3.20 g, 20.5 mmol) in DMF (20 mL) was heated at 80 °C for 10 min by microwave irradiation. The reaction mixture was condensed into a small portion under reduced pressure and then poured into a large amount of ethyl acetate. The precipitates were collected by centrifugation and washed with ethyl acetate several times. The title compound was obtained in 64% yield (391 mg) by the same purification and ion-exchange procedures used to prepare **B1-Br**. ^1^H NMR (DMSO-*d*_6_, 25 °C, see Fig. S1c) *δ:* 1.61 (t, CH_3_, *J* = 7.2 Hz, 3H), 4.73 (q, CH_2_, *J* = 7.2 Hz, 2H), 5.87 (s, N^+^-CH_2_-Ar, 4H), 5.91 (s, N^+^-CH_2_-Ar, 2H), 7.66 (s, Ar-H, 1H), 7.70 (s, Ar-H, 2H), 7.99–8.05 (m, Ar_pyridyl_-H, 4H), 8.63–8.70 (m, Ar_pyridyl_-H, 4H), 8.70–8.81 (m, Ar_pyridinium_-H, 4H), 8.85–8.92 (m, Ar_pyridinium_-H, 4H), 9.19–9.25 (m, Ar_pyridinium_-H, 4H), 9.34–9.41 (m, Ar_pyridinium_-H, 4H).

### Synthesis of B2-Br

The title compound was obtained in 72% yield (230 mg) from **B1-Py** (186 mg, 0.16 mmol) and TBMB (2.22 g, 6.23 mmol) in DMF (1.8 mL) by microwave irradiation at 110 °C for 5 min in the same manner used to prepare **B1-Br**. ^1^H NMR (DMSO-*d*_6_, 25 °C, see Fig. S1d) *δ:* 1.60 (t, CH_3_, *J* = 7.3 Hz, 3H), 4.64–4.77 (s + q, Br-CH_2_ and N^+^-CH_2_, 10H), 5.90 (s, N^+^-CH_2_-Ar, 6H), 5.96 (s, N^+^-CH_2_-Ar, 4H), 7.61 (s, Ar-H, 6H), 7.67 (s, Ar-H, 3H), 8.69–8.82 (m, Ar_pyridinium_-H, 12H), 9.32–9.54 (m, Ar_pyridinium_-H, 12H).

### Synthesis of B2-Py

The title compound was obtained in 88% yield (291 mg) from **B2-Br** (230 mg, 0.11 mmol) and 4,4’-bipyridine (1.41 g, 9.03 mmol) in DMF (9 mL) by microwave irradiation at 80 °C for 10 min in the same manner used to prepare **B1-Py**. ^1^H NMR (DMSO-*d*_6_, 25 °C, see Fig. S1e) *δ:* 1.60 (t, CH_3_, *J* = 7.3 Hz, 3H), 4.73 (q, CH_2_, *J *= 7.2 Hz, 2H), 5.80 (m, N^+^-CH_2_-Ar, 18H), 7.64–7.81 (m, Ar-H, 9H), 7.97–8.04 (m, Ar_pyridyl_-H, 8H), 8.64–8.70 (m, Ar_pyridyl_-H, 8H), 8.70–8.82 (m, Ar_pyridinium_-H, 12H), 8.85–8.92 (m, Ar_pyridinium_-H, 8H), 9.17–9.24 (m, Ar_pyridinium_-H, 8H), 9.31–9.42 (m, Ar_pyridinium_-H, 12H).

### Synthesis of B3-Br

The title compound was obtained in 82% yield (376 mg) from **B2-Py** (291 mg, 0.10 mmol) and TBMB (2.84 g, 7.96 mmol) in DMF (2.4 mL) by microwave irradiation at 110 °C for 5 min in the same manner used to prepare **B1-Br**. ^1^H NMR (DMSO-*d*_6_, 25 °C, see Fig. S1f) *δ:* 1.60 (t, CH_3_, *J* = 7.2 Hz, 3H), 4.64–4.77 (s + q, Br-CH_2_ and N^+^-CH_2_, 18H), 5.85–6.01 (m, N^+^-CH_2_-Ar, 26H), 7.61 (s, Ar-H, 12H), 7.72–7.88 (m, Ar-H, 9H), 8.68–8.84 (m, Ar_pyridinium_-H, 28H), 9.32–9.55 (m, Ar_pyridinium_-H, 28H).

### Synthesis of acetyl-capped B3

The title compound was obtained in 67% yield (134 mg) from **B3-Br** (100 mg, 0.022 mmol) and **AcS-Py** (224 mg, 0.43 mmol) in DMF (0.8 mL) by microwave irradiation at 90 °C for 10 min in the same manner used to prepare **B1-Py**. ^1^H NMR (DMSO-*d*_6_, 25 °C, see Fig. S1g) *δ:* 1.18–1.38 (br, CH_2_, 96H), 1.48 (quint, CH_2_, *J* = 7.3 Hz, 16H), 1.60 (t, CH_3_, *J* = 7.4 Hz, 3H), 1.96 (br, CH_2_, 16H), 2.29 (s, SCOCH_3_, 24H), 2.80 (t, CH_2_, *J* = 7.3 Hz, 16H), 4.62–4.76 (m, N^+^-CH_2_, 18H), 5.80–6.02 (br, N^+^-CH_2_-Ar, 42H), 7.70–7.91 (m, Ar-H, 21H), 8.65–8.84 (m, Ar_pyridinium_-H, 60H), 9.29–9.50 (m, Ar_pyridinium_-H, 60H). ESI-MS: *m*/*z* = 1707.77 [M + 25PF_6_]^5+,^ 1398.98 [M + 24PF_6_]^6+,^ 1178.41 [M + 23PF_6_]^7+,^ 1012.99 [M + 22PF_6_]^8+,^ 884.33 [M + 21PF_6_]^9+,^ 781.41 [M + 20PF_6_]^10+.^ Anal. Calcd for C_311_H_372_F_180_N_30_O_8_P_30_S_8_: C, 40.32; H, 4.05; N, 4.54; S, 2.77. Found: C, 39.82; H, 3.50; N, 4.62; S, 2.66.

### Synthesis of acetyl-capped B1

The title compound was obtained in 69% yield (180 mg) from **B1-Br** (100 mg, 0.14 mmol) and **AcS-Py** (212 mg, 0.41 mmol) in DMF (0.8 mL) by microwave irradiation at 90 °C for 10 min in the same manner used to prepare **B1-Py**. ^1^H NMR (DMSO-*d*_6_, 25 °C) *δ:* 1.16–1.40 (br, CH_2_, 24H), 1.48 (quint, CH_2_, *J* = 7.0 Hz, 4H), 1.60 (t, CH_3_, *J* = 7.2 Hz, 3H), 1.96 (br, CH_2_, 4H), 2.30 (s, SCOCH_3_, 6H), 2.80 (t, CH_2_, *J* = 7.2 Hz, 4H), 4.58–4.80 (m, N^+^-CH_2_, 6H), 5.91 (s, N^+^-CH_2_-Ar, 6H), 7.77 (s, Ar-H, 3H), 8.64–8.88 (m, Ar_pyridinium_-H, 12H), 9.29–9.47 (m, Ar_pyridinium_-H, 12H). ESI-MS: *m*/*z* = 812.23 [M + 4PF_6_]^2+^, 493.17 [M + 3PF_6_]^3+^, 333.64 [M + 2PF_6_]^4+^. Anal. Calcd for C_65_H_84_F_36_N_6_O_2_P_6_S_2_: C, 40.76; H, 4.42; N, 4.39; S, 3.35. Found: C, 40.52; H, 4.35; N, 4.33; S, 3.27.

### Synthesis of acetyl-capped B2

The title compound was obtained in 79% yield (155 mg) from **B2-Br** (92 mg, 0.045 mmol) and **AcS-Py** (233 mg, 0.45 mmol) in DMF (0.8 mL) by microwave irradiation at 90 °C for 10 min in the same manner used to prepare **B1-Py**. ^1^H NMR (DMSO-*d*_6_, 25 °C) *δ:* 1.18–1.37 (br, CH_2_, 48H), 1.48 (quint, CH_2_, *J* = 7.4 Hz, 16H), 1.60 (t, CH_3_, *J* = 7.2 Hz, 3H), 1.97 (br, CH_2_, 8H), 2.30 (s, SCOCH_3_, 12H), 2.80 (t, CH_2_, *J* = 7.2 Hz, 8H), 4.61–4.78 (m, N^+^-CH_2_, 10H), 5.84–6.00 (br, N^+^-CH_2_-Ar, 18H), 7.72–7.83 (m, Ar-H, 9H), 8.68–8.82 (m, Ar_pyridinium_-H, 28H), 9.29–9.48 (m, Ar_pyridinium_-H, 28H). ESI-MS: *m*/*z* = 1309.64 [M + 11PF_6_]^3+,^ 945.99 [M + 10PF_6_]^4+,^ 727.80 [M + 9PF_6_]^5+,^ 582.35 [M + 8PF_6_]^6+,^ 478.45 [M + 7PF_6_]^7+.^ Anal. Calcd for C_147_H_180_F_84_N_14_O_4_P_14_S_4_: C, 40.45; H, 4.16; N, 4.49; S, 2.94. Found: C, 40.25; H, 3.75; N, 4.41; S, 3.02.

### Deprotection of the acetyl-capped molecules

The acetyl group was removed using HBr formed *in situ* by mixing acetyl bromide and methanol according to the method described in the literature^36^,^37^. A typical procedure is described as follows. Acetyl-capped **B3** (100 mg) was dispersed in dehydrated methanol (3 mL). Acetyl bromide was added dropwise to the dispersion at −78 °C under vigorous stirring. After being maintained at −78 °C for 5 min, the reaction mixture was warmed to room temperature and further stirred for 3 h. It was then dried under reduced pressure, the residue was dissolved into methanol, and the resulting solution was then poured into a large amount of ethyl acetate. The ion exchange of Br^-^ for PF_6_^-^ using NH_4_PF_6_ aq. and subsequent purification of the product were performed in a manner similar to that used to prepare **B1-Py**. The thiol-group functionalization of **B3** was confirmed to be approximately 100% on the basis of its NMR spectrum (see Fig. 2a). The obtained **B3** was stored at −80 °C under argon atmosphere. In the same manner, **Vio**, **B1** and **B2** were prepared by deprotection of the corresponding acetyl-capped molecules.

#### Spectroscopic data for **Vio**

^1^H NMR (DMSO-*d*_6_, 25 °C, seeFig. 2a) *δ*: 1.18–1.37 (br, CH_2_, 12H), 1.51 (quint, CH_2_, *J* = 7.4 Hz, 2H), 1.60 (t, CH_3_, *J* = 7.3 Hz, 3H), 1.97 (br, CH_2_, 2H), 2.19 (t, SH, *J* = 7.6 Hz), 2.45 (overlapped with the solvent signal, q, CH_2_, *J* = 7.2 Hz), 4.60–4.76 (q + t, N^+^-CH_2_, 4H), 8.68–8.80 (Ar_pyridinium_-H, 4H), 9.30–9.42 (Ar_pyridinium_-H, 4H). ESI-MS: *m*/*z *= 503.21 [M + PF_6_]^+^, 179.12 [M]^2+^. Anal. Calcd for C_22_H_34_F_12_N_2_P_2_S: C, 40.74; H, 5.28; N, 4.32; S, 4.94. Found: C, 41.02; H, 5.16; N, 4.24, S, 4.73.

#### Spectroscopic data for **B1**

^1^H NMR (DMSO-*d*_6_, 25 °C) *δ:* 1.18-1.40 (br, CH_2_, 24H), 1.51 (quint, CH_2_, *J* = 7.3 Hz, 4H), 1.60 (t, CH_3_, *J* = 7.3 Hz, 3H), 1.97 (br, CH_2_, 4H), 2.19 (t, SH, *J* = 7.7 Hz), 2.45 (overlapped with the solvent signal, q, CH_2_, *J* = 7.2 Hz), 4.60–4.78 (q + t, N^+^-CH_2_, 6H), 5.91 (s, N^+^-CH_2_-Ar, 6H), 7.77 (s, Ar-H, 3H), 8.67–8.85 (Ar_pyridinium_-H, 12H), 9.30–9.44 (Ar_pyridinium_-H, 12H). ESI-MS: *m*/*z* = 770.22 [M + 4PF_6_]^2+,^ 465.16 [M + 3PF_6_]^3+,^ 312.63 [M + 2PF_6_]^4+.^ Anal. Calcd for C_61_H_80_F_36_N_6_P_6_S_2_: C, 40.01; H, 4.40; N, 4.59; S, 3.50. Found: C, 39.46; H, 3.98; N, 4.48, S, 3.42.

#### Spectroscopic data for **B2**

^1^H NMR (DMSO-*d*_6_, 25 °C) *δ:* 1.14–1.41 (br, CH_2_, 48H), 1.51 (quint, CH_2_, *J* = 7.3 Hz, 8H), 1.60 (t, CH_3_, *J* = 7.1 Hz, 3H), 1.97 (br, CH_2_, 8H), 2.19 (t, SH, *J* = 7.8 Hz), 2.45 (overlapped with the solvent signal, q, CH_2_, *J* = 7.2 Hz), 4.58–4.80 (q + t, N^+^-CH_2_, 10H), 5.78–6.04 (m, N^+^-CH_2_-Ar, 18H), 7.66–7.86 (m, Ar-H, 9H), 8.65–8.85 (Ar_pyridinium_-H, 28H), 9.27–9.44 (Ar_pyridinium_-H, 28H). ESI-MS: *m*/*z* = 1253.63 [M + 11PF_6_]^3+,^ 903.99 [M + 10PF_6_]^4+,^ 694.20 [M + 9PF_6_]^5+,^ 554.34 [M + 8PF_6_]^6+,^ 454.44 [M + 7PF_6_]^7+.^ Anal. Calcd for C_139_H_172_F_84_N_14_P_14_S_4_: C, 39.78; H, 4.13; N, 4.67; S, 3.06. Found: C, 39.51; H, 3.91; N, 4.57, S, 3.22.

#### Spectroscopic data for **B3**

^1^H NMR (DMSO-*d*_6_, 25 °C, see Figs. 2a and S1e) *δ*: 1.14–1.41 (br, CH_2_, 96H), 1.51 (quint, CH_2_, *J* = 7.5 Hz, 16H), 1.60 (t, CH_3_, *J* = 7.0 Hz, 3H), 1.97 (br, CH_2_, 16H), 2.19 (t, SH, *J* = 7.7 Hz), 2.45 (overlapped with the solvent signal, q, CH_2_, *J* = 7.2 Hz), 4.60–4.76 (m, N^+^-CH_2_, 18H), 5.80–6.04 (m, N^+^-CH_2_^-^Ar, 42H), 7.66–7.84 (m, Ar-H, 21H), 8.66–8.83 (Ar_pyridinium_-H, 60H), 9.27–9.44 (Ar_pyridinium_-H, 60H). ESI-MS (see Fig. S3): *m*/*z* = 1640.35 [M + 25PF_6_]^5+^, 1342.80 [M + 24PF_6_]^6+^, 1130.26 [M + 23PF_6_]^7+^, 970.86 [M + 22PF_6_]^8+^, ^8^46.88 [M + 21PF_6_]^9+^. Anal. Calcd for C_295_H_356_F_180_N_30_P_30_S_8_: C, 39.69; H, 4.02; N, 4.71; S, 2.87. Found: C, 39.03; H, 3.58; N, 4.72, S, 2.67.

### Synthesis of apical-type dendritic viologen-arranged molecules

The apical-type dendritic molecules were also prepared using **AcS-Py** and **Et-Py** as the starting and capping materials, respectively. Details of the microwave-assisted synthesis of the apical-type molecules will be published elsewhere. The spectroscopic data are described as follows.

#### Spectroscopic data for **A1**

^1^H NMR (DMSO-*d*_6_, 25 °C) *δ:* 1.18–1.38 (br, CH_2_, 12H), 1.51 (quint, CH_2_, *J* = 7.4 Hz, 2H), 1.60 (t, CH_3_, *J* = 7.3 Hz, 6H), 1.97 (br, CH_2_, 2H), 2.20 (t, SH, *J* = 7.7 Hz), 2.45 (overlapped with the solvent signal, q, CH_2_, *J* = 7.2 Hz), 4.63–4.77 (m, N^+^-CH_2_, 6H), 5.91 (s, N^+^-CH_2_-Ar, 6H), 7.78 (s, Ar-H, 3H), 8.69–8.82 (m, Ar_pyridinium_-H, 12H), 9.33–9.43 (m, Ar_pyridinium_-H, 12H). ESI-MS: *m*/*z* = 698.18 [M + 4PF_6_]^2+^, 417.13 [M + 3PF_6_]^3+^. Anal. Calcd for C_53_H_64_F_36_N_6_P_6_S: C, 37.73; H, 3.82; N, 4.98; S, 1.90. Found: C, 37.97; H, 3.61; N, 4.78, S, 2.06.

#### Spectroscopic data for **A2**

^1^H NMR (DMSO-*d*_6_, 25 °C) *δ*: 1.17–1.40 (br, CH_2_, 12H), 1.51 (quint, CH_2_, *J* = 7.3 Hz, 2H), 1.60 (t, CH_3_, *J* = 7.3 Hz, 12H), 1.96 (br, CH_2_, 2H), 2.20 (m, SH), 2.45 (overlapped with the solvent signal, br, CH_2_), 4.58–4.79 (m, N^+^-CH_2_, 10H), 5.80–6.02 (m, N^+^-CH_2_-Ar, 18H), 7.68–7.83 (m, Ar-H, 9H), 8.66–8.86 (m, Ar_pyridinium_-H, 28H), 9.27–9.52 (m, Ar_pyridinium_-H, 28H). ESI-MS: *m*/*z* = 1736.78 [M + 12PF_6_]^2+^, 1109.53 [M + 11PF_6_]^3+^, 795.91 [M + 10PF_6_]^4+^. Anal. Calcd for C_115_H_124_F_84_N_14_P_14_S: C, 36.70; H, 3.32; N, 5.21; S, 0.85. Found: C, 36.83; H, 3.31; N, 5.58, S, 0.96.

#### Spectroscopic data for **A3**

^1^H NMR (DMSO-*d*_6_, 25 °C, see Fig. 2a) *δ*: 1.16–1.42 (br, CH_2_, 1_2_H), 1.48 (br, CH_2_, 2H), 1.60 (t, CH_3_, *J* = 7.3 Hz, 24H), 1.96 (br, CH_2_, 2H), 2.18 (br, SH), 2.80 (overlapped with the solvent signal, br, CH_2_), 4.58–4.82 (m, N^+^-CH_2_, 18H), 5.78–6.02 (m, N^+^-CH_2_^-^Ar, 40H), 7.66–7.83 (m, Ar-H, 21H), 8.64–8.84 (m, Ar_pyridinium_-H, 60H), 9.26–9.46 (m, Ar_pyridinium_-H, 60H). ESI-MS (see Fig. S2): *m*/*z* = 1834.27 [M + 26PF_6_]^4+^, 1438.42 [M + 25PF_6_]^5+^, 1174.53 [M + 24PF_6_]^6+^, 98^6^.03 [M + 23PF_6_]^7+^, 844.67 [M + 22PF_6_]^8+^, 734.70 [M + 21PF_6_]^9+^, 646.72 [M + 20PF_6_]^10+^. Anal. Calcd for C_239_H_244_F_180_N_30_P_30_S: C, 36.26; H, 3.11; N, 5.31; S, 0.40. Found: C, 36.34; H, 2.93; N, 5.54, S, 0.53.

### Preparation of SAMs

Gold substrates were immersed into solutions of the dendritic viologen-arrayed molecules with ω-mercaptodecyl groups in an acetonitrile/ethanol mixture (1/1, v/v) at a concentration of 2 mM under argon atmosphere. After 48 h at room temperature, the resultant SAMs were rinsed with acetonitrile for 10 s and dried under flowing pure nitrogen.

To confirm the chemisorption, a model compound without a mercapto group, *N*-ethyl-*N*′-decyl-4,4′-bipyridinium bishexafluorophosphate (CH_3_(CH_2_)_9_-V^2+^-CH_2_CH_3_/2PF_6_^-^, **C**_**10**_**-V-C**_**2**_) was synthesized and the cyclic voltammogram of a gold substrate treated with the compound in the same manner used to prepare the SAM was measured. No faradaic current was observed in the cyclic voltammogram (Supplementary Fig. S6), which clearly indicates that the redox waves observed in the voltammogram of the SAMs, as shown in Figs 3b and 4a, were due to the viologen units immobilized through the Au-S bonds and that the washing procedure was sufficient to remove free molecules^38^,^39^.

## Additional Information

**How to cite this article**: Kawauchi, T. *et al*. Conical Gradient Junctions of Dendritic Viologen Arrays on Electrodes. *Sci. Rep*. **5**, 11122; doi: 10.1038/srep11122 (2015).

## Supplementary Material

Supplementary Information

## Figures and Tables

**Table 1:** Characterization of SAMs prepared with viologen-arranged molecules.

				_1/2_ *	*Γ*_*V*_ †	*Γ*_*D*_ ‡	*S*_*D*_ ‡	*S*_*P*_ ‡	
Entries	Molecules	*N*_*V*_	*N*_*P*_	mV vs. SCE	unit mol cm^−2^	mol cm^−2^	Å^2^	Å^2^	
1	**Vio**	1	1	−375	4.1 × 10^−10^	4.1 × 10^−10^	40	-	
2	**B1**	3	2	−404	5.0 × 10^−10^	1.7 × 10^−10^	99	50	
3	**B2**	7	4	−406	5.6 × 10^−10^	8.1 × 10^−11^	206	51	
4	**B3**	15	8	−402	6.5 × 10^−10^	4.3 × 10^−11^	385	48	
5	**A1**	3	2	−406	5.3 × 10^−10^	1.8 × 10^−10^	94	47	
6	**A2**	7	4	−394	7.6 × 10^−10^	1.1 × 10^−10^	153	38	
7	**A3**	15	8	−390	9.8 × 10^−10^	0.7 × 10^−10^	253	32	

*N*_V_, number of viologen units. *N*_P_, number of peripheral viologen units. *E*_1/2_, half-wave potential for the V^2+^/V^+•^ process of the viologen units. *Γ*_V_, surface density of viologen unit. *Γ*_D_, surface density of the molecule. *S*_D_, area occupied by one molecule. *S*_P_, area occupied by one peripheral viologen unit.

^*^Determined by CV measurement in 100 mM NaNO_3_.

^†^Determined by chronocoulometry with the potential step from −650 mV to −200 mV vs. SCE in 100 mM NaNO_3_.

^ǂ^Evaluated on the basis of *Γ*_V_.
