# Supplementary material for: Conical Gradient Junctions of Dendritic Viologen Arrays on Electrodes
Source: Sci Rep. 2015 Jun 9;5:11122. doi: 10.1038/srep11122 (PMC4460877; doi:10.1038/srep11122)
Supplement: Supplementary Information [file srep11122-s1.pdf]

<Supplementary Information>

Conical Gradient Junctions of Dendritic Viologen Arrays on Electrodes

Takehiro Kawauchi,* Yuki Oguchi, Keiji Nagai, and Tomokazu Iyoda

Iyoda Supra-Integrated Material Project, Exploratory Research for Advanced Technology (ERATO), Japan Science and Technology Agency (JST), and Frontier Research Center, Tokyo Institute of Technology, 4259-S2-3 Nagatsuta, Midori-ku, Yokohama 226-8503, Japan

Contents

Table S1 | Diffusion coefficients and hydrodynamic radii of acetyl-capped **Vio**, **An**, and **Bn** molecules.

Scheme S1 | Synthetic route of **B3**.

Figure S1 | ^{1}H NMR spectra of the compounds obtained by each reaction step.

Figure S2 | Positive mode ESI-MS spectrum of **A3**.

Figure S3 | Positive mode ESI-MS spectrum of **B3**.

Figure S4 | Optimized structures of acetyl-capped **Vio**, **A1**, **A2**, and **A3** with PF_6^- by using MM calculations.

Figure S5 | Dependence of the cathodic peak current density in CVs of **A3-** and **B3**-SAMs on the scan rate.

Figure S6 | CVs of gold electrodes prepared by immersing into solutions of **Vio** and a model compound without a mercapto group (**C_{10}-V-C_2**).

Table S1 | Diffusion coefficients (*D*s) and hydrodynamic radii (R_Hs) of acetyl-capped Vio, An, and Bn (n = 0-3).*

Entries	Molecules	MW	*D*	R_H
		g mol^{-1}	m^2 s^{-1}	nm
1	**Vio**	691	1.1×10^{-9}	0.52
2	**A1**	1729	6.7×10^{-10}	0.87
3	**A2**	3806	4.7×10^{-10}	1.24
4	**A3**	7960	3.2×10^{-10}	1.81
5	**B1**	1915	6.1×10^{-10}	0.95
6	**B2**	4365	4.1×10^{-10}	1.42
7	**B3**	9264	2.6×10^{-10}	2.25

*The *D*s were determined by NMR measurement in deuterated acetonitrile at 25 ºC. The R_Hs were calculated on the basis of the *D* values according to the Stokes equation.

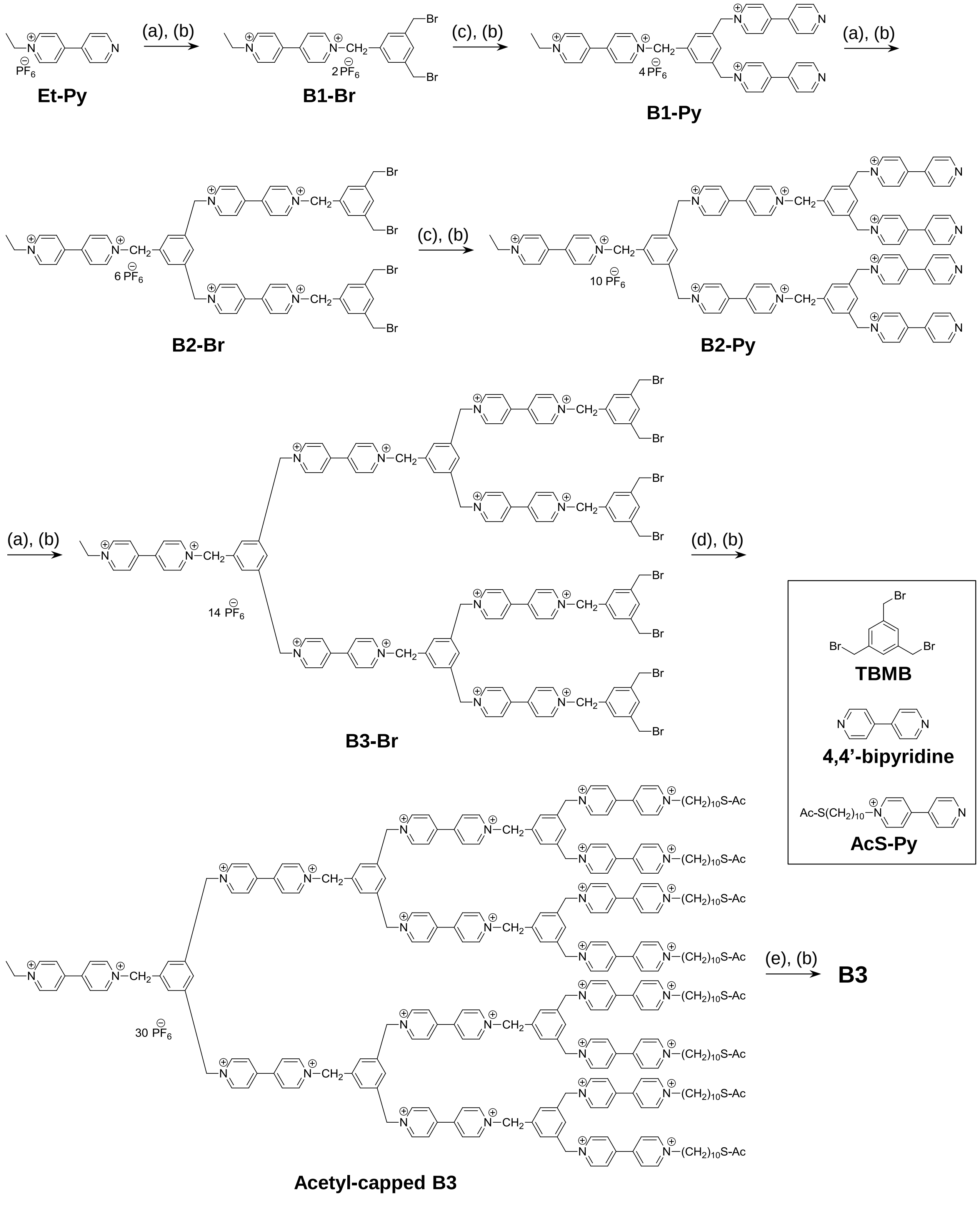

Scheme S1 | Synthetic route of B3. (a) TBMB, microwave, 110 ºC, 5 min. (b) NH_4PF_6 aq. (c) 4,4'-bipyridine, microwave, 80 ºC, 10 min. (d) AcS-Py, microwave, 90 ºC, 10 min. (e) CH_3COBr, CH_3OH.

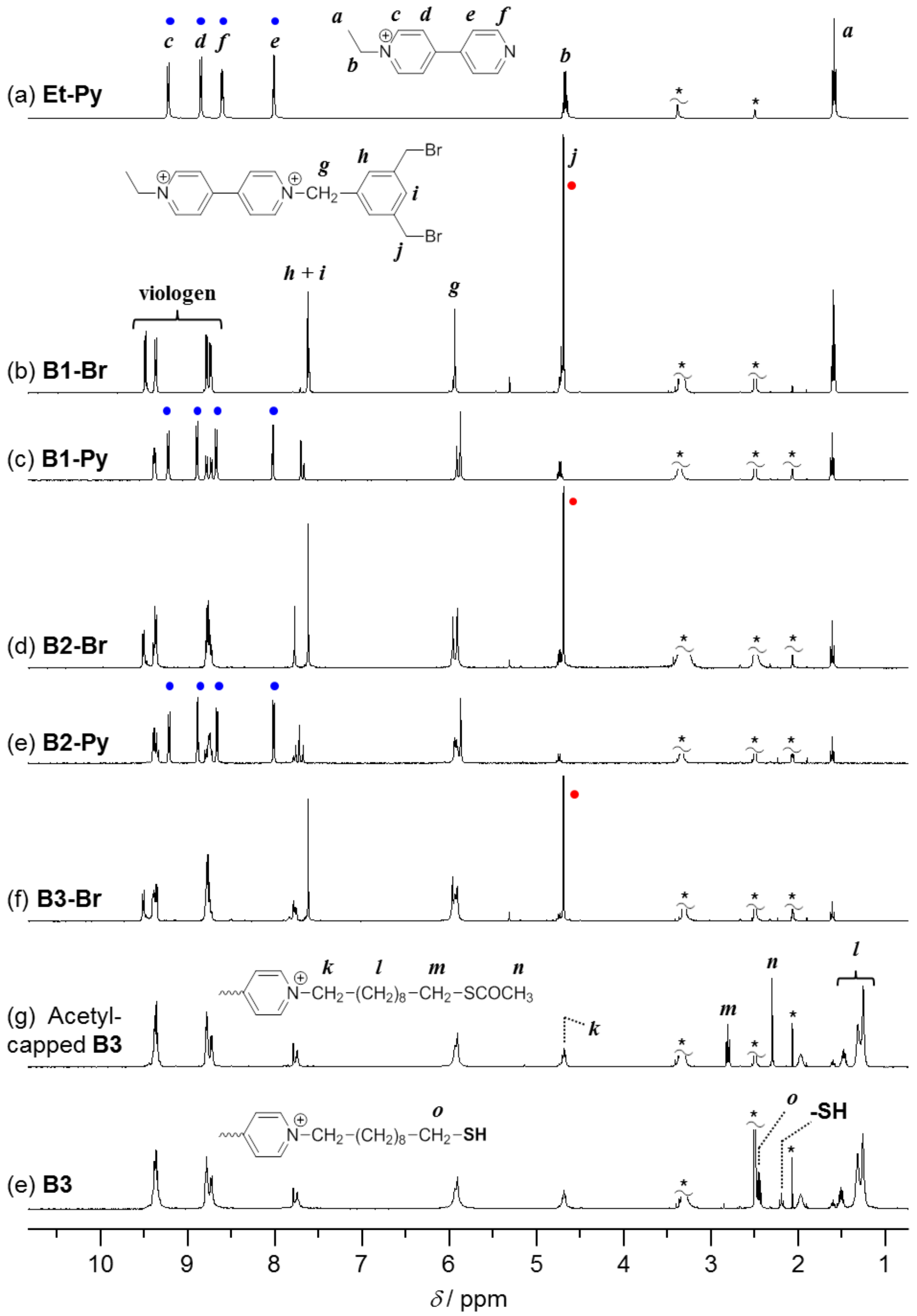

Figure S1 | ¹H NMR spectra of the compounds obtained by each reaction step shown in Scheme S1. The spectra were measured in DMSO-d_6 at room temperature. The asterisks denote CH_3CN (2.06 ppm), DMSO-d_5 (2.49 ppm) and water (3.3 ppm) signals.

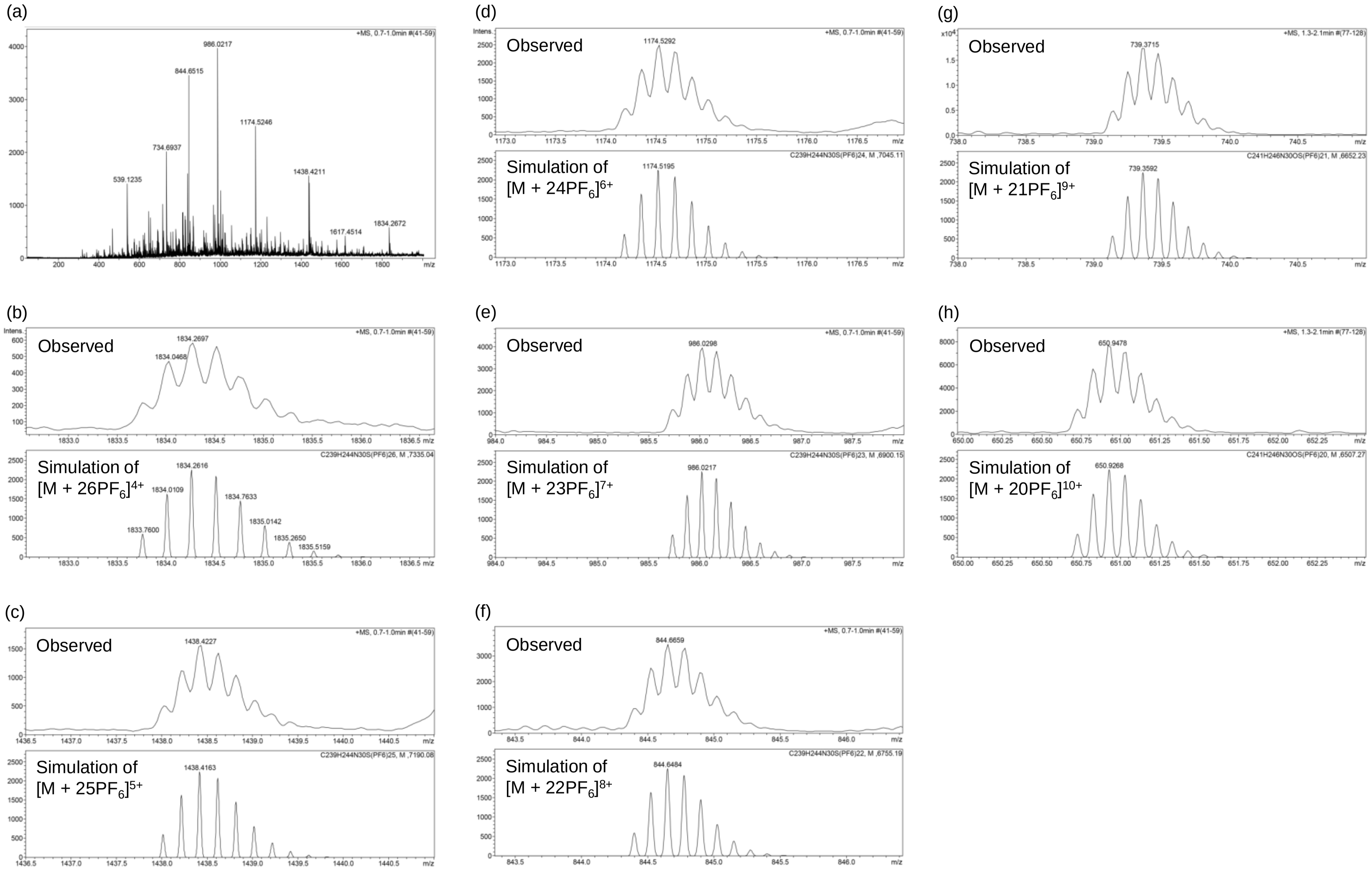

Figure S2 | (a) Positive mode ESI-MS spectrum of **A3**. (b-h) Comparison between the observed peaks (upper) and isotope simulations (bottom).

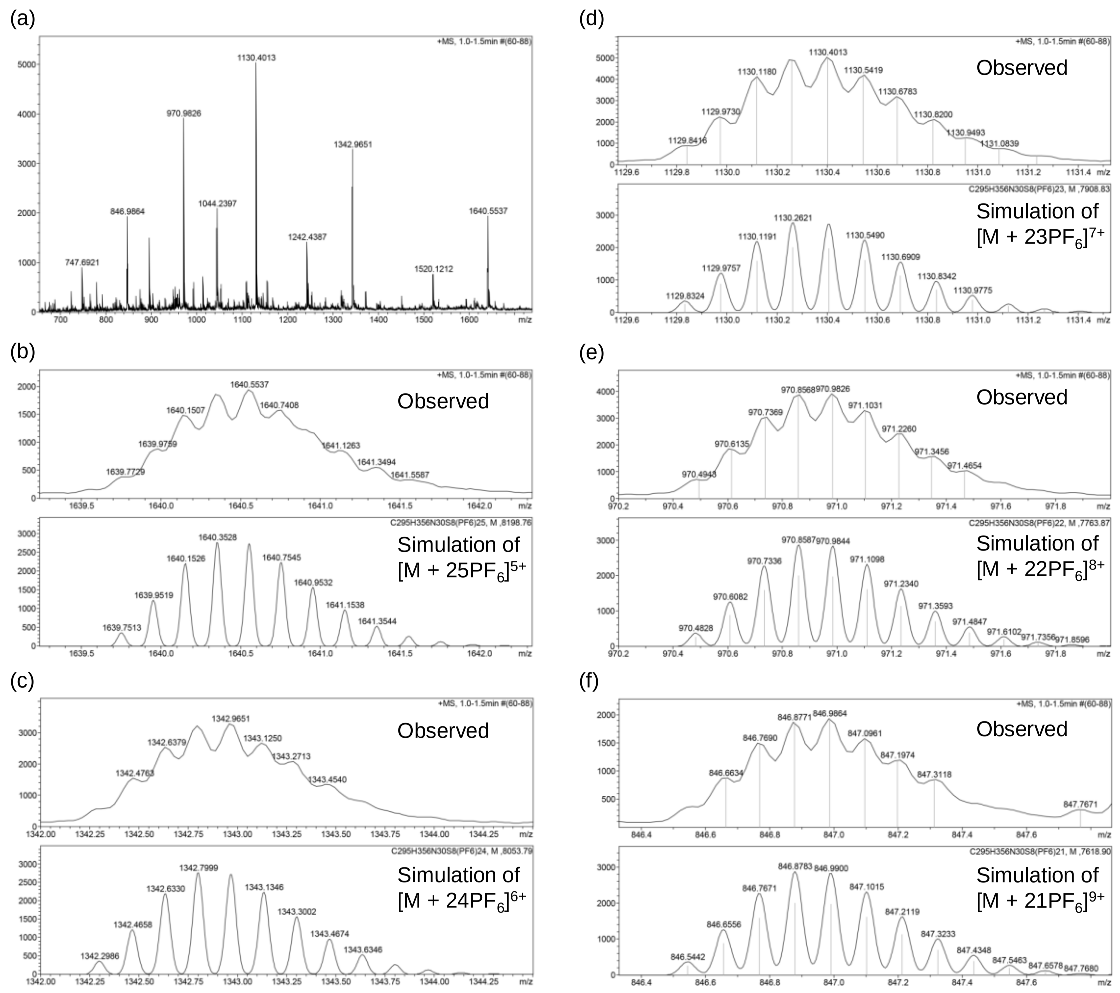

Figure S3 | (a) Positive mode ESI-MS spectrum of **B3**. (b-f) Comparison between the observed peaks (upper) and isotope simulations (bottom).

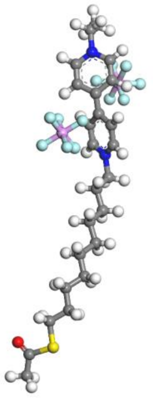

Vio
R_{calc} = 0.73 nm

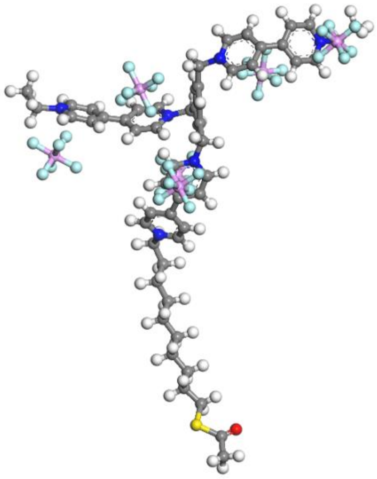

A1
R_{calc} = 0.91 nm

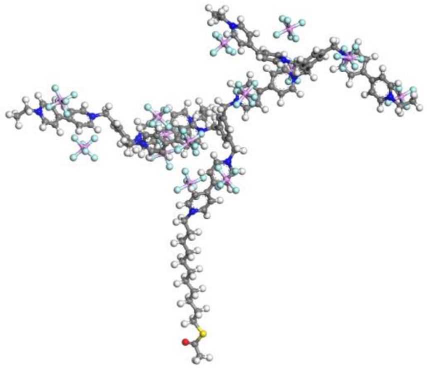

A2
R_{calc} = 1.42 nm

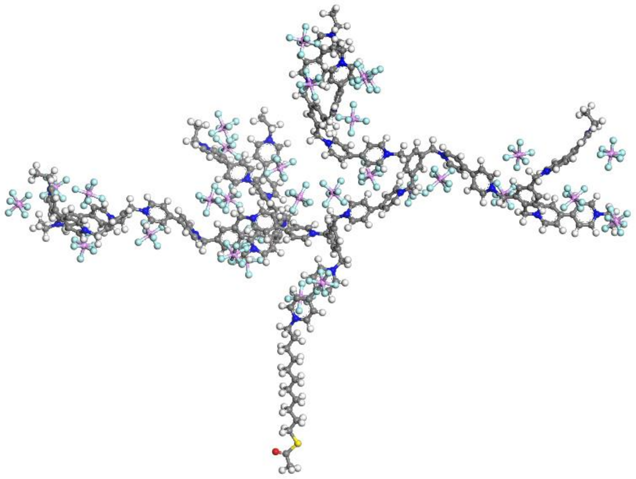

A3
R_{calc} = 2.16 nm

Figure S4 | Optimized structures of acetyl-capped **Vio**, **A1**, **A2**, and **A3** with PF_6^- by using MM calculations with COMPASS-II force field. Radius of gyration (R_{calc}) was estimated using Forcite tool.

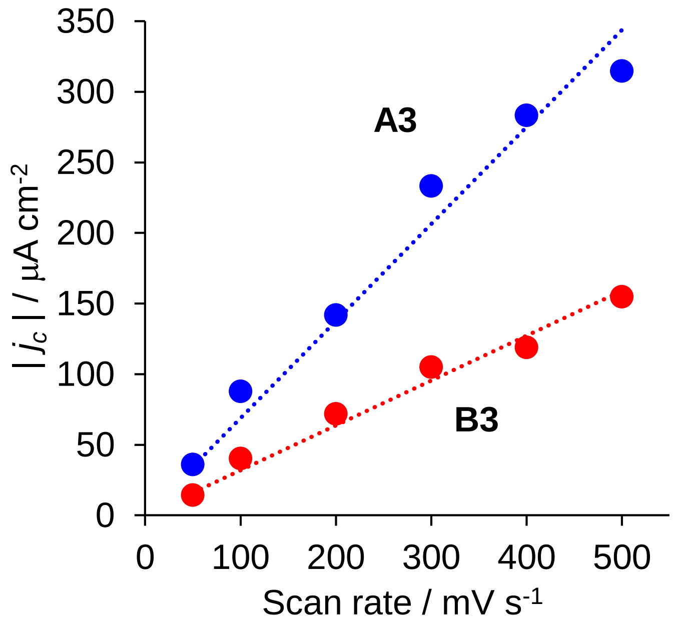

Figure S5 | Dependence of the cathodic peak current density (j_c) in CVs of **A3-** and **B3-**SAMs on the scan rate.

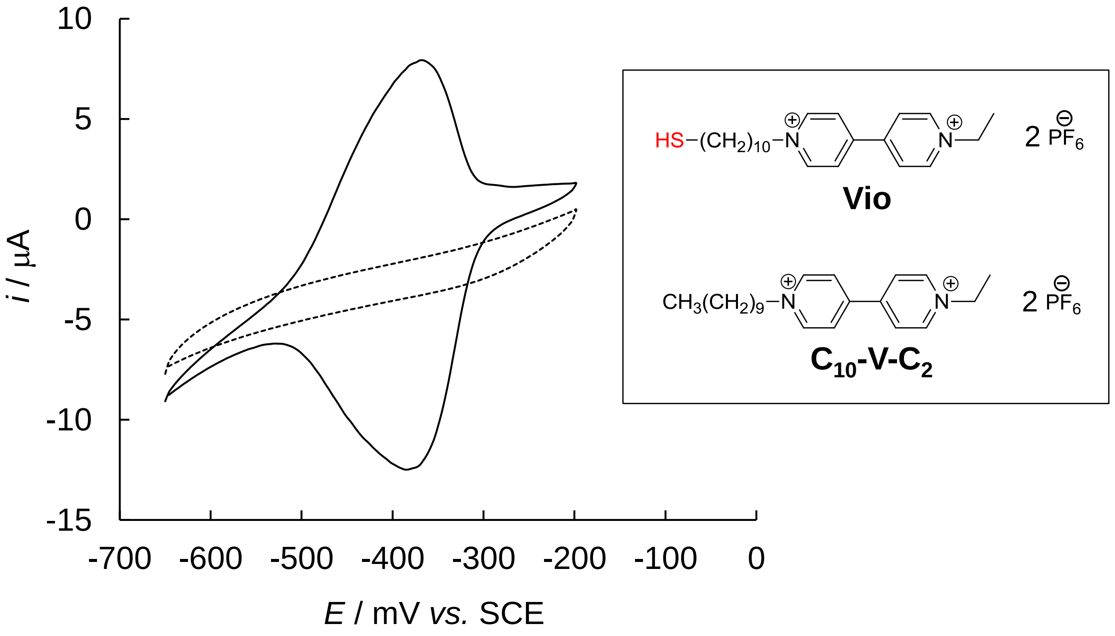

Figure S6 | CVs of gold electrodes prepared by immersing into solutions of **Vio** (solid line) and *N*-ethyl-*N'*-decyl-4,4'-bipyridinium bishexafluorophosphate (**C_{10}-V-C_2**, dotted line) followed by rinsing with acetonitrile and then drying under flowing N_2. The measurement was performed in 100 mM $NaNO_3$ at a scan rate of 200 mV s^{-1}.
